# The safety profile of belzutifan in renal tumors: real-world data from a tertiary academic center

**DOI:** 10.1093/oncolo/oyaf274

**Published:** 2025-09-08

**Authors:** Aaron Jacob Winer, Paulo Siqueira do Amaral, Elizabeth G Ryan, Morgan A Lambrecht, Chiu-Lan Chen, Brian I Rini, Kathryn E Beckermann

**Affiliations:** Department of Medicine, Vanderbilt University Medical Center, Nashville, TN 37232, United States; Vanderbilt-Ingram Cancer Center, Vanderbilt University Medical Center, Nashville, TN 37232, United States; Vanderbilt-Ingram Cancer Center, Vanderbilt University Medical Center, Nashville, TN 37232, United States; Vanderbilt-Ingram Cancer Center, Vanderbilt University Medical Center, Nashville, TN 37232, United States; Department of Biostatics, Vanderbilt University Medical Center, Nashville, TN 37232, United States; Vanderbilt-Ingram Cancer Center, Vanderbilt University Medical Center, Nashville, TN 37232, United States; Vanderbilt-Ingram Cancer Center, Vanderbilt University Medical Center, Nashville, TN 37232, United States

**Keywords:** belzutifan, VHL, spRCC, anemia, hypoxia, safety

## Abstract

**Background:**

Belzutifan is a HIF-2ɑ inhibitor approved for the treatment of tumors in von Hippel-Lindau (VHL) syndrome and sporadic metastatic clear cell renal cell carcinoma (spRCC) in the refractory setting. The efficacy and side effects of belzutifan are well-documented from clinical trials; however, real-world data examining the incidence and management of adverse events (AEs) are lacking. Our study aims to describe the AE profiles of belzutifan in spRCC and VHL populations.

**Methods:**

A retrospective analysis was conducted at Vanderbilt University Medical Center assessing patients who received belzutifan monotherapy. Primary endpoints were the incidence of anemia and hypoxia. Secondary endpoints included time to onset of anemia and hypoxia, as well as management strategies.

**Results:**

Forty-four patients were identified with either spRCC (*n* = 22) or VHL syndrome (*n* = 22). Patients with spRCC were older than VHL patients (median 67 vs 41 years) and had higher rates of chronic kidney disease (36.4% vs 4.5%) and prior nephrectomy (77.3% vs 40.9%). The spRCC patients had a median follow-up time of 3.8 months vs 26.8 months in VHL patients. Any-grade anemia occurred in the majority of spRCC and VHL patients (81% and 95.5%, respectively) with a median time of 25 days in spRCC patients and 77 days in VHL patients. While no patient with VHL experienced grade 3 anemia, 41% of spRCC patients developed grade ≥3 anemia. In spRCC, grade ≥3 hypoxia developed in 54.5% and for VHL patients, grade 3 hypoxia occurred in 9%. Median time to grade ≥3 hypoxia was 29 days (range 12-123) in spRCC patients and 225 days (range 105-345) in VHL patients. Supplemental oxygen was required in 52.5% of spRCC patients and 9.5% in VHL patients. Treatment discontinuation due to AEs occurred in 50% of spRCC patients and 13.6% of VHL patients.

**Conclusions:**

The time to onset and severity of belzutifan AEs may differ between patients with VHL syndrome and spRCC. These findings suggest the need for a patient-centered approach to monitor and manage toxicity based on disease setting.

Implications for Practice While belzutifan’s efficacy and side effect profile have been defined in clinical trials, data regarding its safety remain limited in the real-world setting. Our retrospective study provides a detailed analysis of adverse events (AEs) associated with belzutifan in both VHL and spRCC populations. This manuscript demonstrates differences observed in the incidence, timing, and severity of anemia between the 2 populations. Our findings suggest that patients with spRCC may be at higher risk for early and severe AEs, often resulting in treatment interruption and discontinuation. These results highlight the need for clearer guidelines for disease-specific toxicity monitoring and management.

## Introduction

The VHL-HIF-VEGF pathway is a compelling therapeutic target for both von Hippel-Lindau (VHL) syndrome and sporadic clear cell renal cell carcinoma (spRCC) patients, given the high prevalence of *VHL* mutations or alterations in these conditions. Germline pathogenic mutations drive VHL syndrome, while in spRCC, *VHL* mutations or hypermethylation occur in approximately 90% of spRCC cases, leading to dysregulation of the hypoxia-inducible factor (HIF) pathway.^1–4^ Under normal physiologic conditions, the VHL protein facilitates the degradation of HIF-2α, preventing the activation of hypoxia-related genes such as erythropoietin (EPO) and vascular endothelial growth factor (VEGF).^5^ However, loss of *VHL* function leads to HIF-2α accumulation and the promotion of tumorigenesis through downstream pathways of angiogenesis, cell cycle regulation, and altered metabolic dependency.

Belzutifan, a selective HIF-2α inhibitor, marked a significant advance in the treatment of VHL-associated tumors and spRCC. In these patients, belzutifan exerts antitumoral effects by binding HIF-2α, thereby inhibiting downstream transcriptional activity.^6^ Beyond its role in oncogenesis, HIF-2α also regulates EPO production in renal interstitial cells. As a result, HIF-2α inhibition suppresses EPO synthesis, causing anemia, a well-documented on-target adverse effect (AE) of belzutifan.^6^^,^^7^ Hypoxia has emerged as another AE of interest. While the precise mechanisms remain unclear, HIF-2α is thought to have a role in the pulmonary vasculature and carotid body, and its blockage may affect cardiopulmonary response to hypoxia.^8^^,^^9^ Belzutifan may alter the ability of normal vasculature having constrictive response to hypoxia, thus worsening ventilation/perfusion mismatch and hypoxia.^8^ Similarly, in the carotid body, an increased respiratory drive in response to detected hypoxia may be blocked by belzutifan, blunting the normal respiratory drive to dropping oxygen levels.^8^

The LITESPARK-004 and LITESPARK-005 trials demonstrated the clinical efficacy and safety of belzutifan in VHL disease and spRCC.^10^^,^^11^ In LITESPARK-004, which evaluated belzutifan in patients with VHL syndrome-associated neoplasms, the objective response rate (ORR) was 49% in renal lesions, 77% in pancreatic lesions, and 30% in central nervous system (CNS) hemangioblastomas.^10^ The safety profile demonstrated grade 1/2 anemia in 90% of patients with grade ≥ 3 anemia occurring in only 8%. In patients with VHL syndrome, hypoxia occurred in only one patient.^10^ LITESPARK-005, which randomized patients with refractory spRCC to belzutifan vs everolimus, demonstrated that belzutifan led to higher ORR of 21.5% vs 3.5% and improved 18-month progression-free survival rates of 24% vs 8.3% with everolimus.^11^ While low-grade anemia remained common (82.8%), higher rates of grade ≥ 3 anemia and grade ≥ 3 hypoxia were observed in spRCC patients in LITESPARK-005,^11^ occurring in 32.5% and 10.5% of patients, respectively. Although both trials demonstrated comparable efficacy, the differences in serious AE remain unexplained.

As belzutifan was recently approved in both VHL disease and spRCC, there is a need to better understand the AE profile of these patient populations in everyday practice. This study aims to enhance the understanding of its AE profile and contribute to the optimization of treatment in VHL syndrome and spRCC.

## Methods

### Eligibility criteria

Subjects were eligible for inclusion if they were ≥ 18 years of age with either sporadic renal cell carcinoma or VHL syndrome with an associated tumor. Patients must have received belzutifan as a single agent at any disease stage or line of therapy. Patients were excluded if they received belzutifan as part of combination therapy or were lost to follow-up. Patients were included if they had a minimum follow-up time of 1.5 months.

### Data collection

Patients were identified using a pharmacy database query of all belzutifan prescriptions at Vanderbilt University Medical Center (VUMC) between August 30, 2021 and August 30, 2024. Data were also collected using SlicerDicer, a data exploration tool proprietary to the Epic health record system that allows for customized searches among VUMC patients. Using SlicerDicer, subjects were identified who had been prescribed belzutifan over a 5-year period. This study was conducted under IRB approval #160979.

### Assessment

Demographic and clinical data were collected from medical records. The primary endpoint was the incidence of anemia and hypoxia classified as any grade and grade ≥ 3, per Common Terminology Criteria for Adverse Events (CTCAE 5.0). Secondary endpoints included time to anemia, need for supplemental ESA and/or blood transfusion, and dose reduction, treatment interruption, discontinuation, or hospitalization. Similar outcomes were measured for hypoxia, including time to hypoxia, need for supplemental oxygen therapy, and hypoxia-related belzutifan dose reduction, treatment interruption, or discontinuation. The endpoints above were evaluated separately for the spRCC and VHL syndrome cohorts.

### Time intervals

The median time to events was determined from the date of belzutifan initiation until the event of interest. These events included anemia and hypoxia.

## Results

### Patient characteristics

A total of 44 patients who received belzutifan monotherapy were identified. Twenty-two patients had spRCC, and 22 patients had VHL-associated tumors, including bilateral RCC (45.5% of patients), unilateral RCC (22.7%), CNS hemangioblastoma (86.4%), retinal hemangioma (59.1%), and pancreatic neuroendocrine tumor (50%) (Table 1).

**Table 1.** VHL disease manifestations. VHL, von Hippel-Lindau.

VHL disease manifestations	
**RCC**	15 (68.2)
** Unilateral RCC**	5 (22.7)
** Bilateral RCC**	10 (45.5)
**CNS hemangioblastoma**	19 (86.4)
**Pancreatic neuroendocrine tumor**	11 (50.0)
**Ear endolymph**	2 (9.1)
**Pheochromocytoma**	6 (27.3)
**Retinal hemangioma**	13 (59.1)
**Paraganglioma**	2 (9.1)

Abbreviations: CNS, central nervous system; RCC, renal cell carcinoma; VHL, Von Hippel-Lindau.

The VHL cohort had a younger age distribution than the spRCC cohort, with a median age of 41 years (IQR 33-54), compared to a median age of 67 years (IQR 62-71) (Table 2). The VHL group had a female predominance (63.6%), whereas spRCC patients were predominantly male (68.2%). At treatment initiation, Eastern Cooperative Oncology Group (ECOG) performance status was 0 or 1 in all patients, except for one VHL patient and 2 spRCC patients, who had ECOG scores of 2. SpRCC patients more frequently had chronic kidney disease (CKD) (36.4% vs 4.5%) and a higher nephrectomy rate (77.3% vs 40.9%). Other baseline characteristics such as comorbidities, baseline supplemental oxygen use, and smoking history were similar between groups (Table 2). Baseline hemoglobin (Hgb) values were similar between groups: the VHL cohort had a median Hgb of 13 g/dL (IQR 12-15), while the spRCC cohort had a median of 13 g/dL (IQR 10-14).

**Table 2.** Patient characteristics.

	VHL patients (*n* = 22)	Sporadic RCC (*n* = 22)
**Age**		
** Median (IQR)**	41 (33-54)	67 (62-71)
**Sex, *n* (%)**		
** Male**	8 (36.4)	15 (68.2)
** Female**	14 (63.6)	7 (31.8)
**ECOG, *n* (%)**		
** 0-1**	21 (95.5)	20 (90.9)
** 2**	1 (4.5)	2 (9.1)
**Comorbidities, *n* (%)**		
** Smoking history**	9 (40.9)	12 (54.6)
** Obesity**	11 (50.0)	10 (45.5)
** CKD (estimated Glomerular Filtration Rate (eGFR)** **< 50)**	1 (4.5)	8 (36.4)
** COPD**	0 (0)	3 (13.6)
** Prior venous thromboembolism (VTE)**	1 (4.5)	2 (9.1)
** Baseline supplemental O_2_**	0 (0)	2 (9.1)
**Prior nephrectomy**	9 (40.9)	17 (77.3)
**Baseline hemoglobin, g/dL**		
** Median (IQR)**	13 (12-15)	13 (10-14)
** Mean ± SD**	14 ± 2	12 ± 3
**Time to follow-up, months**		
** Median (IQR)**	27 (21-29)	4 (2-5)
**Duration on belzutifan, months**	31 (22-33)	3 (2-6)
** Median (IQR)**		
**Number of metastatic sites, median (range)**	0	2 (1-4)
**Sites of metastasis**		
** Lung**	0	20 (90.9)
** Lymph node**	0	9 (40.9)
** Bone**	0	9 (40.9)
** Adrenal gland**	0	5 (22.7)
** Liver**	0	3 (13.6)
**≥3 sites of metastatic disease, no. (%)**	N/A	8 (36.4%)
**Number of lines of therapy, median (range)**	N/A	3 (1-7)
**Line of therapy for belzutifan, no. (%)**		
** 2 L**		4 (18.2)
** 3 L**		5 (22.7)
** ≥4 L**		13 (59.1)
**Prior treatment before belzutifan start, no. (%)**		
** ICI**		21 (95.5)
** Tyrosine kinase inhibitor (TKI)**		21 (95.5)

Abbreviations: ECOG, Eastern Cooperative Oncology Group; ICI, immune checkpoint inhibitor; VHL, Von Hippel-Lindau.

Time to follow-up, defined as the duration between the first dose of belzutifan and last treatment follow-up, was longer in VHL patients (median of 27 months [IQR 21-29]) compared to spRCC patients (median of 4 months [IQR 2-5]). Duration on belzutifan therapy was similarly longer in the VHL cohort (median of 31 months [IQR 22-33]) vs the spRCC cohort (median of 3 months [IQR 2-6]). Of spRCC patients, 36.4% of patients had 3 or more sites of metastatic disease, with the lung (90.9%), lymph node (40.9%), bone (40.9%), adrenal (22.7%), and liver (13.6%) being the most common. SpRCC patients had a median of 3 prior lines of therapy (range 1-7). In spRCC patients, 95.5% had previously received immune checkpoint inhibitors (ICIs), with ICI combinations being the most common prior line of therapy (50% of patients).

### Adverse events

**Figure 1.**
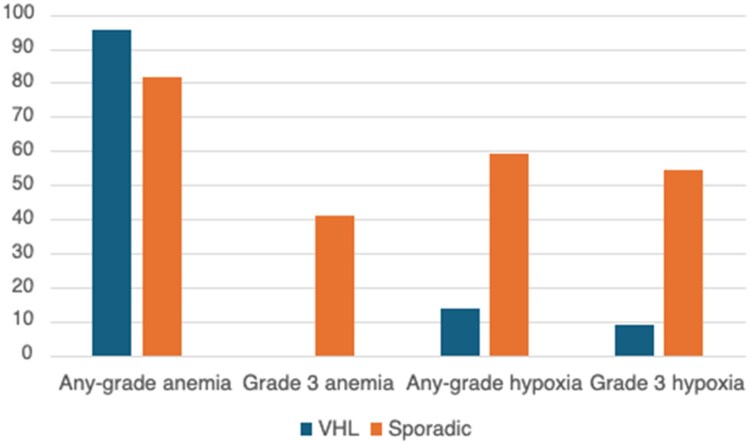
Incidence of anemia and hypoxia (%).

**Figure 2.**
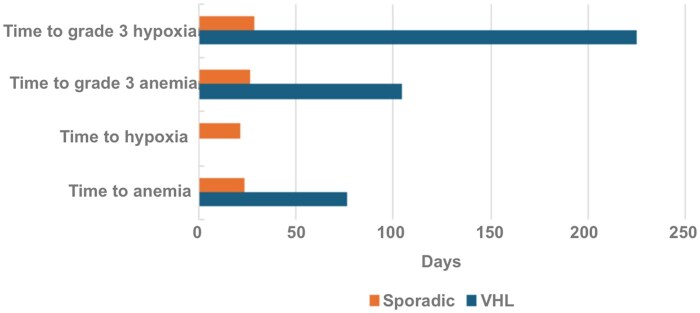
Time to AE development. AE, adverse event.

In spRCC patients, 81.8% of patients had any-grade anemia and 41% of patients developed grade 3 anemia (Figure 1); time to onset was 24 days (range 14-40) and 22 days (range 21-43), respectively (Figure 2). For hypoxia, any-grade hypoxia occurred in 59% of spRCC patients, while grade 3 hypoxia occurred at a rate of 54.5% (Figure 1); time to onset was 27 days (range 21-41) and 29 days (range 21-47), respectively (Figure 2). No deaths attributed to treatment occurred in spRCC patients.

In the VHL cohort, 95.5% of patients developed any-grade anemia with a median time to onset of 77 days (range 49-278). Grade 3 anemia did not occur. Any-grade hypoxia occurred in 13.6% of patients; median time to onset was 105 days (range 84-225). Grade 3 hypoxia occurred in 9.1% of VHL patients with a median time to onset of 225 days (range 165-285). Death occurred in one VHL patient from an unknown cause during follow-up.

### Management of AEs

**Figure 3.**
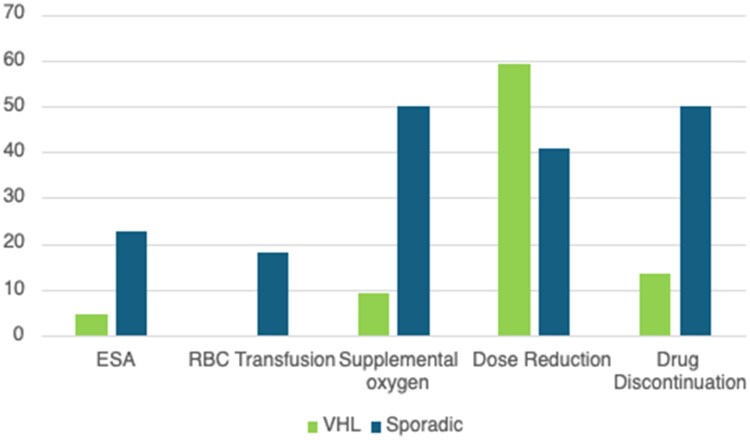
AE management (%). AE, adverse event.

The management of anemia and hypoxia was assessed among the entire 44-patient cohort (Figure 3). Of patients who developed anemia, 5 (11.4%) received erythropoietin-stimulating agents (ESAs) only, 3 (6.8%) received blood transfusions only, and one (2.3%) received both ESA and a blood transfusion. Fourteen patients (31.8%) had their belzutifan held due to anemia, and 9 of these patients (20.5%) had dose reductions. The median time to resolution of anemia was 28 days (range 1-434). For hypoxia, 13 patients (29.5%) required supplemental oxygen, and 11 patients (25%) were hospitalized due to hypoxia. Of the patients who developed hypoxia, 4 patients required supplemental oxygen of ≥ 6 liters, 3 patients required ICU-level care, and 2 patients were intubated due to acute hypoxic respiratory failure. In total, half of the entire study population required dose reduction in treatment, thirty-one patients (70.5%) required treatment interruption, and treatment was permanently discontinued in 14 patients (31.8%).

Measures for AE management were next assessed in each group. In spRCC patients, management included ESA alone in 4 patients (18.2% of patients), blood transfusions alone in 3 patients (13.6%), and both ESA and blood transfusions in one patient (4.5%). Eleven spRCC patients (50%) were treated with supplemental oxygen. In spRCC, treatment modification due to AE included belzutifan dose reduction in 9 patients (40.9% of patients), treatment interruption in 16 (72.7%), and treatment discontinuation in 11 patients (50%) (Table 3). Of these 11 patients who discontinued treatment, 7 spRCC patients discontinued treatment due to intolerance, the causes of which included hypoxia in 5 patients and both anemia and hypoxia in 2 patients. The remaining 4 patients discontinued treatment due to disease progression (PD).

**Table 3.** Dose reduction, treatment interruption, discontinuation.

	Pooled population (*n* = 44)	VHL (*n* = 22)	Sporadic RCC (*n* = 22)
**All cause dose reduction, *n* (%)**	22 (50.0)	13 (59.1)	9 (40.9)
**All cause treatment interruption, *n* (%)**	31 (70.5)	15 (68.2)	16 (72.7)
**Treatment discontinuation, *n* (%)**	14 (31.8)	3 (13.6)	11 (50.0)
** Tolerance**	8	1	7
** Anemia**	1	1	0
** Hypoxia**	5	0	5
** Both anemia and hypoxia**	2	0	2
** Disease progression**	5	1	4
** Death**	1	1	0

Abbreviations: RCC, renal cell carcinoma; VHL, Von Hippel-Lindau.

In VHL patients, one patient received ESA only, and no VHL patients required blood transfusions. Two patients (9.1%) received supplemental oxygen for hypoxia, and no patients required hospitalization during the management of their hypoxia. Due to AEs, 59.1% had dose reduction, 68.2% treatment interruption, and 13.6% treatment discontinuation. Of the 3 VHL patients who discontinued treatment, the causes included one case of anemia, one case of PD, and one patient had passed away as previously noted.

## Discussion

In this retrospective analysis of 44 patients treated with belzutifan, there were no newly emergent AEs beyond those reported in the clinical trials that led to drug approval. However, in this real-world population, we observed that patients with spRCC developed high-grade anemia and hypoxia more frequently and rapidly. Among the 22 spRCC patients, high-grade anemia and hypoxia occurred in nearly half of the patients. In contrast, the 22 patients in our series with VHL had no grade ≥3 anemia and only one case of grade ≥3 hypoxia, consistent with the findings of LITESPARK-004.^10^

Several factors may have contributed to the high AE burden in our spRCC group vs the prospective trials LITESPARK-001, -005, and -013,^9^^,^^11^^,^^12^ where grade ≥3 anemia rates were 19.7%-32.5% and hypoxia ranged from 10.5%-21.1%. Our group was more heavily pre-treated, with 60% of our spRCC patients having received belzutifan as the ≥ fourth line of therapy, compared to less than 1% of LITESPARK-005 patients. Additionally, patients treated in the real-world setting more commonly have a lower performance status and comorbidities that might exclude patients from being optimal candidates for clinical trial participation.

Similarly, the contrast in AE rates between our spRCC and VHL cohorts may be explained by differences, including age, comorbidities (eg, CKD, chronic obstructive pulmonary disease (COPD), fewer drugs for other diseases), and a higher tumor burden. We observed a lower glomerular filtration rate in the spRCC cohort which is associated with loss of EPO-producing interstitial cells, which may contribute to the lower mean hemoglobin.^13^^,^^14^ The higher rate of prior nephrectomy in the spRCC group may further contribute to anemia, given the kidney’s known role in EPO production. The higher rate of hypoxia in this real-world spRCC compared to the LITESPARK or our VHL patients may in part be related to impaired ventilation-perfusion mismatch either through the presence of lung metastasis in spRCC patients (90.9%), the presence of pulmonary embolism, or other underlying comorbidities such as COPD, obesity, or heart failure.

In VHL patients, management strategies of anemia and hypoxia were not drastically different from LITESPARK-004. Dose reductions due to any AE occurred at a higher frequency in our VHL population (59.1 vs 15%), but AE-related treatment discontinuation rarely occurred in both our population and the prospective trial (4.5 vs 2%), reinforcing the tolerability of belzutifan in VHL patients, even in the real-world setting. Compared with spRCC patients, VHL patients required fewer interventions for anemia and hypoxia as well as all-cause treatment discontinuation.

For both anemia and hypoxia in our spRCC patients, dose reduction and treatment interruption occurred at a higher rate than the prospective studies, which is likely a reflection of the higher rate of severe AEs in our spRCC patients. Most of our patients required treatment interruption for hypoxia, underscoring the need for close follow-up in real-world patients, as most AEs develop within 3 to 4 weeks of treatment initiation.^15^ Regarding management of anemia in our spRCC patients, we found that use of ESA and blood administration was similar to LITESPARK-005 and -013 (16.7%-23.9% and 13.4%-18%, respectively). The variability in ESA use is in part related to differing trial protocols and The Food and Drug Administration (FDA) label, which currently offers no guidance on ESA use in the context of HIF inhibition.

Currently, there is a paucity of guidelines for AE monitoring and management in patients receiving belzutifan. These data prompt the further need to document real-world use of this novel class of drug and create guidelines for HIF inhibitor toxicity management. Considerations include baseline and intermittent assessments with pulmonary function testing, arterial blood gas with A-a gradient calculation, or ventilation-perfusion scintigraphy. Patient education is necessary regarding monitoring and reporting common side effects via home O_2_ pulse oximeters and monitoring for declining vital signs. Hypoxia management is relatively standardized, with recommendations for holding belzutifan and initiating supplemental oxygen use in exertional or resting hypoxia (O_2_ ≤ 88%). In contrast, anemia management lacks standardized thresholds for intervention. The FDA label does not support ESA use due to insufficient safety and efficacy data. Monitoring frequency is also undefined. Our data support closer monitoring in the first 3–4 weeks of therapy, when most AEs arise. Prior studies demonstrate peak EPO suppression occurs around 2 weeks into belzutifan therapy, suggesting hemoglobin should be checked at this time, especially in patients with baseline hemoglobin <12 g/dL.^15^

Pharmacogenomic variation may also contribute to toxicity risk. Belzutifan exposure-response analyses show an increased risk of grade 3 anemia in patients with poor metabolizer genotypes of UGT2B17 and CYP2C19.^7^ However, a phase 1 dose escalation study meant to recapitulate levels of belzutifan seen in intermediate and poor metabolizers did not show any safety concerns.^16^ Although the FDA label advises monitoring dual poor metabolizers, it remains unclear which patients warrant testing. Poor metabolizer prevalence for CYP2C19 is up to 2%–6% in Caucasians and 20% in Asians.^17^

Limitations of this study include differences in follow-up intervals, which may have introduced detection bias. Additionally, spRCC patients underwent more frequent clinical and laboratory evaluations than VHL patients who often had longer follow-up overall. These differences, along with the retrospective nature and limited sample size of our study, are important limitations to acknowledge. A multivariable analysis would help disentangle the independent effects of baseline characteristics, but our limited sample size and retrospective design precluded the approach; future larger cohorts with sufficient power will be necessary to perform these analyses.

## Conclusion

This analysis identified important differences in both the incidence and time to onset of anemia and hypoxia between patients with spRCC and those with VHL syndrome, as well as between real-world spRCC patients and those enrolled on the registrational clinical trials. These findings underscore the need for a personalized approach to monitoring and managing patients taking belzutifan. Further studies are needed to help develop guidelines regarding follow-up strategies and toxicity management to best optimize patient outcomes.

## Data Availability

The data underlying this article will be shared on reasonable request to the corresponding author.
